# Editorial: Training Methodology: A Multidimensional Approach for Team Sports

**DOI:** 10.3389/fpsyg.2022.862465

**Published:** 2022-03-16

**Authors:** Ana Filipa Silva, José Afonso, Hugo Sarmento, Daniel Castillo, Gibson Moreira Praça, Javier Raya-González, Luca Paolo Ardigò, Rodrigo Aquino, Rodrigo Ramirez-Campillo, Beat Knechtle, Filipe Manuel Clemente

**Affiliations:** ^1^Escola Superior Desporto e Lazer, Instituto Politécnico de Viana do Castelo, Rua Escola Industrial e Comercial de Nun'Álvares, Viana do Castelo, Portugal; ^2^The Research Centre in Sports Sciences, Health Sciences and Human Development (CIDESD), Vila Real, Portugal; ^3^Research Center in Sports Performance, Recreation, Innovation and Technology (SPRINT), Melgaço, Portugal; ^4^Centre for Research, Education, Innovation and Intervention in Sport, Faculty of Sport of the University of Porto, Porto, Portugal; ^5^University of Coimbra, Faculty of Sport Sciences and Physical Education, Research Unit for Sport and Physical Activity, Coimbra, Portugal; ^6^Faculty of Education, Universidad de Valladolid, Soria, Spain; ^7^Sports Department, Universidade Federal de Minas Gerais, Belo Horizonte, Brazil; ^8^Faculty of Health Sciences, University Isabel I, Burgos, Spain; ^9^Department of Neurosciences, Biomedicine and Movement Sciences, School of Exercise and Sport Science, University of Verona, Verona, Italy; ^10^Department of Sports, Center of Physical Education and Sports, Federal University of Espírito Santo, Vitória, Brazil; ^11^Exercise and Rehabilitation Sciences Laboratory, Faculty of Rehabilitation Sciences, School of Physical Therapy, Universidad Andres Bello, Santiago, Chile; ^12^Institute of Primary Care, University of Zurich, Zurich, Switzerland; ^13^Medbase St. Gallen Am Vadianplatz, Gallen, Switzerland; ^14^Instituto de Telecomunicações, Delegação da Covilhã, Lisboa, Portugal

**Keywords:** sports training, training methodology, sports psychology, athletic performance, sports sciences

The theory and methodology of training combine different factors that support the coach's intervention for maximizing the athlete's performance. Among these factors can be included the testing and monitoring, the definition of targets and structure of intervention, the planning, and the intervention itself or in a larger concept a hybrid model factor supporting performance as recovery strategies, psychological interventions, nutrition, or supplementation.

Since performance is multidimensional, it seems interesting to look for this issue from a different perspective, namely considering interaction among factors and providing reports about those interactions. This was the main rationale for supporting the opening of this Frontiers topic.

Overall, we have published 12 articles in our topic. Different approaches were received, as expected. Although the range of topics of research, it was obvious two major areas in which the articles were focused: (i) routes for integrating psychology into the sports training methodology; and (ii) testing and Monitoring into the sports training methodology.

Among those included in the “routes for integrating psychology into the sports training methodology,” it was observed articles were more related with a description of psychological factors, while others were more focused on identifying how to use training interventions by using the psychology background.

Regarding the articles related to testing and monitoring, it was obvious a specific concern in quantifying and qualifying the training intensities and the wellbeing of athletes across the season. Additionally, the characterization of specific exercises, tasks, or interventions was also focused on.

Considering the interest of evidence presented in our topic, following the readers can briefly overview the multitude of topics and the main findings reported in the included articles.

## Routes for Integrating Psychology Into the Sports Training Methodology

The research on sport personality traits has increased exponentially over the past decade. However, some aspects need to be better understood, and the study of Piepiora analyzed assessed in a detailed way the personality traits affecting men's performance in team sports. Based on a sample of 300 players from team sports, the authors concluded that there exist differences between team sports in four personality traits, namely: neuroticism, extraversion, agreeableness, and conscientiousness. The author concluded that the distribution levels of personality traits depend on the sport discipline. In this sense, champions of team sports seem to be characterized by: (1) a lower level of neuroticism; (2) a higher level of extraversion, and (3) openness to experiences in relation to other sportsmen. Consequently, an important role must be assigned to those mental training techniques that favor emotional balance, team communication, and tactical thinking skills and are manifested in triggering start-up readiness.

The scientific evidence on fear in competitive athletes is minimal. However, the investigation in extreme sport provides an understanding of how individuals experience fear in high-risk sports. The study of Rogers and Paskevich provides valuable information concerning the Canadian national team (Alpine Ski) men's experience and management of fear. Through a qualitative analysis the authors concluded that one's experience and management of fear may be influenced by confidence and contextual factors. The discrepancy between the athletes' approaches to training and racing, making it difficult to master fear management strategies. Based on those conclusions the authors presented a set of practical recommendation that can be applied by both coaches and sport psychologists.

Bergmann et al. performed a systematic review investigating the influence of practice design and coaching behavior on perceptual-motor and perceptual-cognitive skill acquisition in soccer. Nearly half the studies (*n* = 18) focused on instructional approaches such as Differential Learning, Teaching Games for Understanding and so-called Non-linear Pedagogy. The other half of the studies (*n* = 17) investigated task design and coaches' instructions, but did not follow a specific instructional philosophy, model, or approach. Considerable heterogeneity was found among the studies in both categories (e.g., study design, participants, interventions, comparators, and outcomes), and despite data supporting the use of pedagogical approaches that promote autonomy and self-exploration, more so-called “traditional” approaches also generated improvements in technical outcomes, but their effects on tactical parameters were scarcely explored. Furthermore, most studies had small samples, lacked consideration of relevant confounders, and the average Downs and Black score was merely 55.65%. We support the authors' conclusions that research on instructional models would benefit from more high-quality studies, with randomized designs and, preferably, blinded outcome assessors. As the authors stated, coaches should comprehensively analyze the context and the participants before choosing a specific pedagogical approach, as a “one-size-fits-all” approach is unlikely to exist.

One study translated knowledge and insights from the preparation of the Belgian special forces operators into elite team sports training (Pattyn et al.). The demands for very high performances in contexts of uncertainty and under time pressure, the need to balance respect for the team structure and goals with the requirement of making creative, spontaneous, individual decisions, often needing out-of-the-box solutions, and the year-long demand of readiness for performing are features common to special forces and elite team sports training. The bar is set very high for psychological and physical characteristics, and only a small percentage of candidates achieves the required levels of performance. The authors recommend an integrated development of physical and psychological skills and propose that exercise for prevention of injuries and exercise for rehabilitation share many features. Centered on evolving from a dualistic mind-body thinking to an integrate framework of training processes, the authors propose a bold approach to simultaneously develop both the individual and the team.

In the study of Klatt et al. with elite 46 males and 36 females German beach volleyball players, it was asked to fill out the Big Five Inventory, the Personality Adjective Scale, and the Affective Style Questionnaire. The aim was to assess the personality traits and emotion regulation styles of those athletes. In general, the same personality traits and values were shared among players, i.e., they exhibited a similar profile of personality. That profile includes a higher manifestation of warmth, liveliness, emotional stability, and reasoning, along with lower levels of neuroticism in successful athletes. The players used a variety of emotional regulation styles and reported being moderately to highly satisfied with their team. In fact, neuroticism was the most observed profile, rather than extraversion, agreeableness, and conscientiousness, which proved to be little observed. In general, beach volleyball players can be characterized as spontaneous and lively. This study seems to provide more information for coaches, sport psychologists, and academics for practical application and further scientific research.

In a systematic review with meta-analysis, the authors (Macías et al.) aimed to understand if using conventional or non-conventional sport teaching methodologies could influence students' enjoyment/fun. Eleven studies were included in this analysis. Despite the great heterogeneity of the included studies, the interventions that conducted non-conventional teaching methods showed significant improvements, exhibiting a moderate effect size (0.72, and a 95% CI from 0.48 to 0.96). It seems that the methodology used by teachers plays an important role, with the Sport Education Model showing the highest indices of students' enjoyment/fun. On the other hand, the traditional methodology was not able to promote the enjoyment/fun of boys and girls in sports practice. These suggestions could be useful for teachers and sport coaches to implement during sport practice. Nevertheless, more studies are needed in this field as the GRADE analysis recognized a low quality of the evidence.

## Testing and Monitoring Into the Sports Training Methodology

The participation in a soccer match can lead acute and residual fatigue, inducing a decline in physical performance and increase levels of muscle damage (e.g., creatine kinase activities—Ck) over the following hours and days. In addition, soccer matches can affect the running performance indicators (e.g., total distance covered in different speed thresholds), muscle damage and fatigue differently depending of the playing positions. Therefore, Freire et al. determined the impact of soccer matches on Ck activities, recovery responses and the specific Global Positioning System (GPS)-accelerometry-derived performance analysis according to playing positions (i.e., defenders, offensive midfielders, forwards, wingers, strickers). Twenty-four professional soccer teams of the Brazilian League Serie A participated of the study. Blood Ck activities were measured pre-, immediately post-, and 24 h postmatch, and the GPS-accelerometry parameters were assessed during the matches. The main results demonstrated that Ck responses were higher in all post-match time points compared to pre-match. Furthermore, recovery markers were also identified up to 24 h after the matches, especially for midfielders. The study showed higher values of running performance indicators (e.g., total distance, total load, sprint frequencies above 18 km·h^−1^) in international competitions in South America than at the state level in Rio de Janeiro/Brazil. These results provide new insigths into the information of a specific time-course recovery for each playing positions after elite professional soccer matches. Consequently, coaches and practitioners would adopt a position specific recovery program after the soccer match, particularly for midfielders who are exposed to higher muscle damage after the soccer matches.

Considering the ultimate goal at sports performance is achieving the highest performance to compete, another factor for attending real concern is the wellbeing state of athletes. Specifically, the distribution of training within- and between-weeks from the wellbeing state perspective is crucial to understand the preparation process of athletes for competition. As such, we can find the article of Nobari et al., in which the authors carried out a descriptive–longitudinal study across a full-season (i.e., 36 weeks) for a soccer team. Players completed the Hooper questionnaire after each training session, allowing authors to analyze the weekly variations of wellbeing status relative to fatigue, stress, delayed-onset muscle soreness and sleep quality. Also, Nobari et al. considered to describe these wellbeing variables attending to playing positions (i.e., goalkeeper, fullback, center half, center midfielder, winger, and forward) and moments of the season (i.e., pre-, early-, mid-, and end-season). Main results reported higher fluctuations in wellbeing status indicators at the beginning and at the end of the season in comparison to the middle one. Also, the differences found in some wellbeing variables between training days and match-play were significant, so we can remark the importance of recovery up to 48 h after a match. Finally, these results about wellbeing variations across the season could help coach staffs to make decisions preventing higher risk of injuries.

Training methodology in team sports accounts for various factors that possibly impact players' performance. Besides the training loads, which are extensively investigated in the literature, an interesting factor to be taken into account is the surface on which the training is prescribed. Specifically in soccer, besides the higher specificity of the grass regarding the demands of the competition, the prescription of training sessions in the sand surface has been previously suggested. However, there is no consensus in the literature on this topic. For this reason, Cetolin et al. compared the internal responses of soccer players to high-intensity exercises performed on the sand and the grass. The study recruited nine U-23 soccer players for a randomized repeated measures design in which the players performed a high-intensity intermittent exercise (HIIE) session in both surfaces interspersed by 48 h to reduce the fatigue effect. The oxygen consumption (VO_2_), blood lactate concentration and rating of perceived exertion were measured to compare the players' responses between the surfaces. The results, in general, indicated that performing HIIE on sand elicits a higher internal workload in terms of cardiorespiratory, metabolic, and perceptual responses compared to the same exercise performed on the grass. This result outlines the potential of sand-based activities to improve the VO2peak and aerobic running performance.

These authors (Torres-Banduc et al.) assessed female volleyball players for jump performance (jump height, contact time, and reactive strength index), concomitant to eccentric and concentric phase surface electromyography (gastrocnemius medialis, biceps femoris, and vastus medialis muscles), during drop jumps from different drop heights (15–90 cm). Jump performance variables were not significantly (*p* > 0.05) different between drop heights. Moreover, the mean electromyography ranged from 27 to 120% of maximal voluntary isometric contraction, although without significant differences between drop heights. Therefore, jumping performance and most neuromuscular markers were not sensitive to drop jump height (intensity), suggesting that lower drop heights may induce similar training stimulus compared to higher drop heights.

Hamstring complex is the muscle group with the highest incidence in soccer. These injuries are considered as multifactorial, being the hamstring flexibility a relevant risk factor. In this sense, Abate Daga et al. analyzed the hamstring flexibility rate among prepubertal Italian soccer players attending to the age and soccer years of practice. Six-hundred and fourteen outfield soccer players (U8 = 124 players; U9 = 130 players; U10 = 151 players; U11 = 89 players; and U12 = 120 players) from a soccer academy in the city of Turin, Italy, were recruited for this study. Authors used the Sit and Reach Test to assess hamstring flexibility and showed significant differences in flexibility among groups. Specifically, significant differences between U8 and U10, U8 and U11, U8 and U12, U9 and U12, U10 and U12, U11 and U12 were observed. Additionally, a negative association between the age categories and hamstrings flexibility was found, being similar for body mass index. Due to the observed differences in hamstring flexibility across age groups of prepubertal soccer players, it is necessary to implement individualized stretching protocols to reduce the risk of injuries derived from an excess of hamstring tightness.

Nikolaidis et al. focused on the relationship between the force-velocity (F-v) test and the Wingate anaerobic test (WAnT) players on a cycle ergometer in 158 regional-level male soccer. They aimed at finding (experimental group, EXP, *n* = 79) and validating (control, CON, *n* = 79) two prediction equations of absolute (Ppeak [W]) and mass-normalized [rPpeak (W·kg^−1^)] power as a function of F-v test results. Once theoretical maximal force [F_0_ (N)], maximal absolute [P_max_ (W)] and mass-normalized [rP_max_ (W·kg^−1^)] power and maximal velocity [v_0_ (rpm)] were calculated from F-v test, prediction equations resulted: P_peak_ = 44.251 + 7.431·body mass [kg]+0.576·P_max_-19.512·F_0_ (*R*^2^ = 0.833) and rP_peak_ = 3.148 + 0.218·rP_max_ + v_0_ (*R*^2^ = 0.585). Equations' validations did not show any significant bias between predicted and true values neither for P_peak_ (*p* = 0.661), nor for rP_peak_ (*p* = 0.525). Overall, F-v test can be suggested as a valid alternative to more fatiguing WAnT, especially when the trainer is interested in the team's average power more than in individual values.

## Author Contributions

All authors made equal work participating in the writing, revision, and approval of the article.

## Conflict of Interest

The authors declare that the research was conducted in the absence of any commercial or financial relationships that could be construed as a potential conflict of interest.

## Publisher's Note

All claims expressed in this article are solely those of the authors and do not necessarily represent those of their affiliated organizations, or those of the publisher, the editors and the reviewers. Any product that may be evaluated in this article, or claim that may be made by its manufacturer, is not guaranteed or endorsed by the publisher.

